# Early Presentation and Management of Normal Pressure Hydrocephalus in a Middle-Aged Patient: A Case Report

**DOI:** 10.7759/cureus.89838

**Published:** 2025-08-11

**Authors:** Chukwuemeka E Ogbu, Shoon L Oo, Anjali Gupta, Jagroop Doad, Maureen Ezechukwu, Yasier Medina, Elizabeth Onyeaso

**Affiliations:** 1 Internal Medicine, Cape Fear Valley Health, Fayetteville, USA; 2 Medicine, Campbell University School of Osteopathic Medicine, Lillington, USA; 3 Neurology, Cape Fear Valley Health, Fayetteville, USA

**Keywords:** gait disturbance, idiopathic nph, middle-aged adult, neurology and neurosurgery, normal-pressure hydrocephalus

## Abstract

Normal pressure hydrocephalus (NPH) is an uncommon neurological disorder that is most often seen in adults over 60 years of age. Its pathogenesis remains uncertain but is believed to involve impaired cerebrospinal fluid (CSF) absorption, increased resistance to CSF outflow, or a mismatch between CSF production and resorption. Reports of NPH in patients younger than 60 are rare, as advanced age is closely associated with the condition. We describe a 55-year-old woman who presented to the emergency department with progressive ataxia, cognitive slowing, and urinary incontinence. Brain imaging demonstrated ventriculomegaly consistent with NPH, and she underwent continuous lumbar drainage with marked clinical improvement. She subsequently participated in an inpatient rehabilitation program, which resulted in further gains in mobility and cognition. Our case highlights the importance of considering NPH in middle-aged patients presenting with compatible symptoms and imaging findings, as timely intervention can lead to meaningful recovery.

## Introduction

Normal pressure hydrocephalus (NPH) is a potentially reversible etiology of dementia arising from the accumulation of cerebrospinal fluid (CSF), subsequently resulting in enlarged ventricles [1]. Under normal physiology, CSF is produced in the choroid plexus, circulates through the ventricular system and subarachnoid space, and is reabsorbed into the bloodstream via arachnoid granulations. In NPH, this balance is disrupted either through impaired reabsorption or altered resistance to flow, leading to ventricular dilation without elevated intracranial pressure [1, 2]. Since it was first clinically described in 1965, NPH has been classically associated with the symptomatic triad of dementia, urinary incontinence, and gait ataxia [2]. The cause of NPH may be multifactorial, including those related to congenital defects or vascular abnormalities [3]. However, it is generally divided into idiopathic normal pressure hydrocephalus (iNPH) and secondary normal pressure hydrocephalus (sNPH) [1]. iNPH is often observed in adults older than 60 years and makes up 80-90% of cases, while sNPH can occur at any age and can occur due to a wide variety of etiologies such as intracranial, infectious, or traumatic [1]. Epidemiologically, NPH affects approximately 3.7% of individuals over the age of 65, while its prevalence in those under 60 is estimated to be less than 0.01% therefore making it a diagnostic challenge in younger adults [4].

The diagnosis of NPH can be challenging due to the presence of associated comorbidities and normal age-related functional or cognitive decline [4]. Nonetheless, its presentation is typically slow and progressive, and can involve postural instability, heterogeneous gait disturbances, cognitive impairment, and uninhibited neurogenic bladder [4]. Imaging can often provide ancillary information with computed tomography (CT) showing ventriculomegaly (defined using the Evans Index, a ratio of the width of the frontal horns of the lateral ventricles to the widest internal diameter of the skull; a value >0.3 is suggestive of NPH) and magnetic resonance imaging (MRI) [5]. The gold standard treatment of NPH is with CSF shunting procedures such as ventriculoperitoneal, ventriculopleural, or ventriculoatrial shunting [2]. However, prior to these procedures, a lumbar drainage test known as the "tap test" which is a diagnostic procedure in which 30-50 mL of CSF is removed through lumbar puncture to assess for clinical improvement or continuous lumbar drainage test can be beneficial to assess effects of CSF removal on symptoms, especially gait derangements, as well as presurgical evaluation prior to shunt placement [6].

Here, we present a case that is particularly notable given the patient's age, as NPH predominantly affects individuals over 60 years old, with limited cases reported in the literature involving younger patients. A 55-year-old female presented to the emergency department with several months of progressively worsening vertigo, gait ataxia, confusion, and urinary incontinence. A CT scan revealed ventricular enlargement, and with her constellation of symptoms, she was diagnosed with NPH. A therapeutic lumbar puncture (LP) was performed, with rapid improvement in the patient's symptoms, and she was seen by the physical therapy team for rehabilitation.

## Case presentation

This patient is a 55-year-old female with a past medical history of attention deficit hyperactivity disorder, generalized anxiety disorder, depression, gastroesophageal reflux disease, hyperlipidemia, and hypertension who presented to the emergency department (ED) with persistent lightheadedness, progressive gait instability, nausea, episodes of confusion, and urinary incontinence. Her husband reported that these symptoms had worsened gradually over several months, with urinary incontinence beginning the day before presentation. The incontinence was occasionally accompanied by vomiting or coughing. She denied fever, abdominal pain, diarrhea, or constipation.

On arrival, she was afebrile and hemodynamically stable (heart rate 81 beats/min, respiratory rate 20 breaths/min, blood pressure 135/95 mmHg). She required assistance to ambulate and could not complete formal gait testing, such as the Timed Up and Go (TUG) test, due to instability and fall risk. No focal motor or sensory deficits were identified. Although formal cognitive testing was not performed in the ED or on admission, multiple provider notes documented deficits in attention, short-term memory impairment, and inability to retain or recall information during conversations.

Upon admission, the patient's labs are shown below (Table 1).

**Table 1:** Admission laboratory values with corresponding reference ranges

Variable	Result	Unit	Reference range
Sodium	144	mmol/L	135–145 mmol/L
Potassium	3.8	mmol/L	3.5–5.0 mmol/L
Anion gap	18	mmol/L	8–16 mmol/L (may vary)
White blood cell count (WBC)	9.9	x10³/µL	4.0–10.5 x10³/µL
Hemoglobin	14.9	g/dL	13.5–17.5 g/dL (male) / 12.0–16.0 g/dL (female)
Hematocrit	44.7	%	38.8–50.0% (male) / 34.9–44.5% (female)
Platelets	334	x10³/µL	150–400 x10³/µL

Her nausea and vomiting were thought to be secondary to normal pressure hydrocephalus (NPH) and were managed with ondansetron 4mg every six hours and a scopolamine patch to be changed every 72 hours.

**Figure 1:**
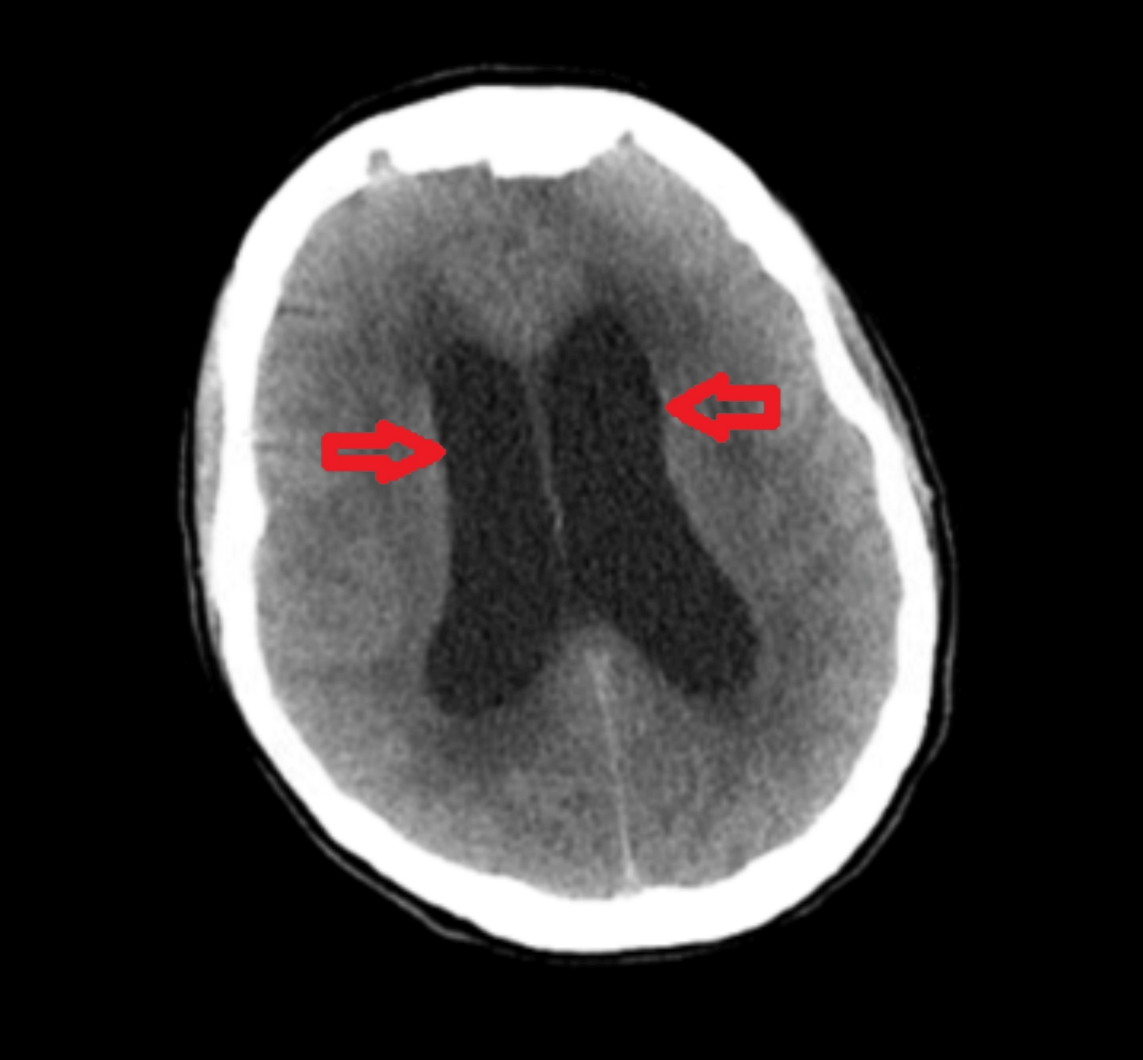
Axial non-contrast CT head showing presence of disproportionate ventricular enlargement and scattered periventricular hypoattenuation

Non-contrast computed tomography (CT) of the head revealed disproportionate ventricular enlargement with periventricular hypoattenuation, suggesting transependymal CSF flow. There were no acute intracranial hemorrhages, mass lesions, or fractures (Figure 1).

**Figure 2:**
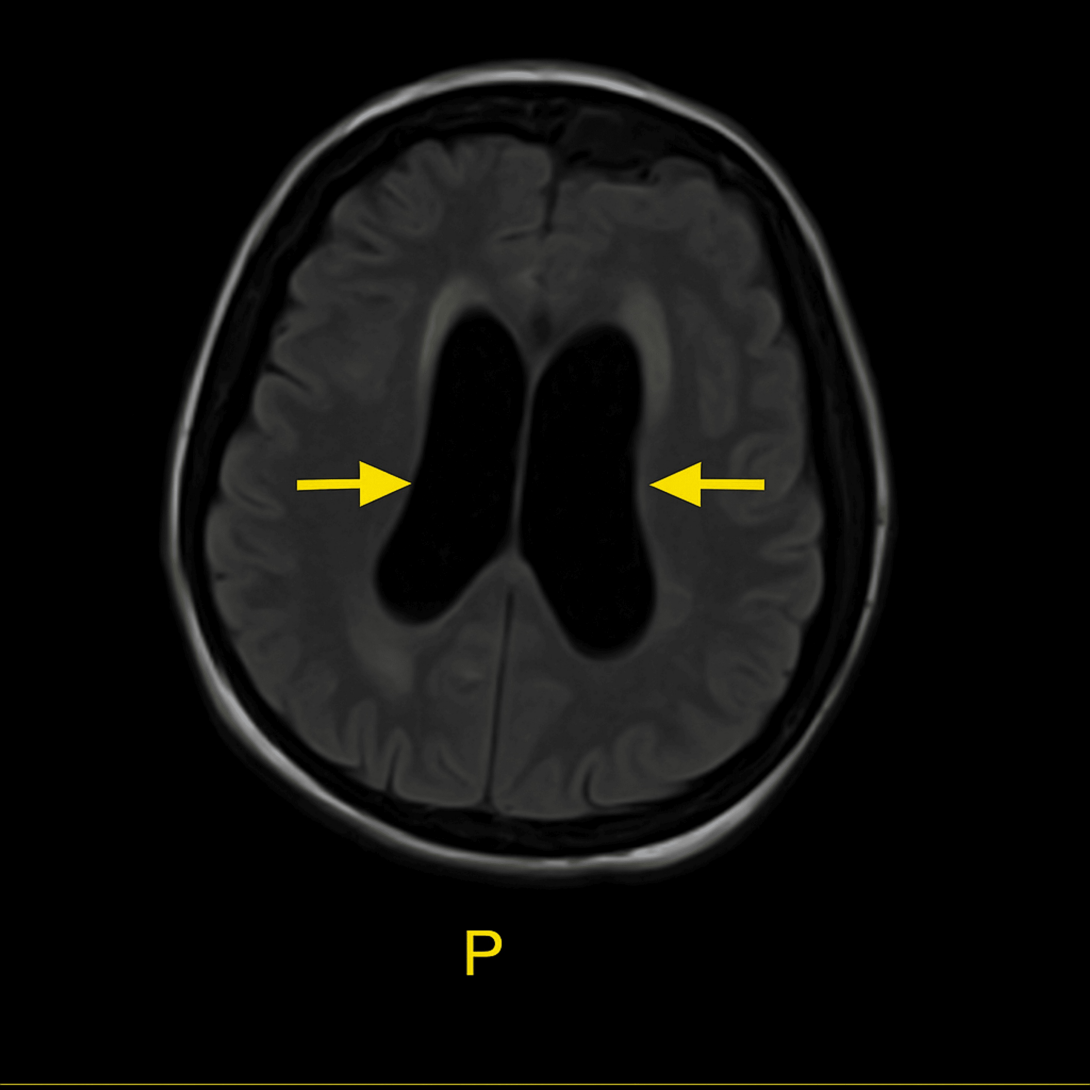
Axial T2-weighted FLAIR MRI demonstrating dilated lateral ventricles (arrows) consistent with ventriculomegaly FLAIR - fluid-attenuated inversion recovery

At this time, neurosurgery was consulted and recommended magnetic resonance imaging (MRI), which demonstrated hydrocephalus and findings concerning for transependymal flow of CSF (Figure 2).

Neurosurgical evaluation suggested a large volume lumbar puncture (LP) to aid in diagnosis and rule out or establish NPH as the cause. The procedure was performed under sterile conditions with the patient in the left lateral decubitus position. Clear cerebrospinal fluid (CSF) was obtained, and an initial 10 mL sample was sent for analysis.

Because the patient's symptoms did not significantly improve immediately after the LP, but NPH remained strongly suspected, neurosurgery proceeded with lumbar drain placement for extended CSF diversion. The patient was placed in the left lateral decubitus position with gentle sedation using Dilaudid and Ativan. The lumbar spine was surgically prepped, and a 140 Tuohy needle was inserted into the L3-L4 interspace with clear CSF visualized. Lumbar drain was inserted to 20cm, needle and style removed, and connected to the Becker drainage system under sterile technique. Drainage was initiated at 10-20 mL every two hours.

CSF analysis was unremarkable for meningitis and was negative for both gram stain and venereal disease research laboratory (VDRL) (Table 2).

**Table 2:** Cerebrospinal fluid analysis results Abnormal values are bolded. The elevated protein, myelin basic protein, and cell counts, though nonspecific, were interpreted as reactive. Infectious etiologies were excluded by negative cultures and meningitis/encephalitis panel CSF - cerebrospinal fluid

CSF parameter	Patient value	Reference range
Glucose	22 mg/dL ↓	40 – 70 mg/dL
Protein	88 mg/dL ↑	15 – 45 mg/dL
Red blood cell count (RBC)	550 cells/μL ↑	0 – 5 cells/μL
Total nucleated cell count	95 cells/μL ↑	0 – 5 cells/μL
Appearance	Slightly Cloudy	Clear
Myelin basic protein	8.6 ng/mL ↑	0 – 3.7 ng/mL
Culture	Negative	Negative
Meningitis/encephalitis panel	Negative	Negative

There were no significant overnight events following the initiation of continuous lumbar drainage. Within 12 hours, the patient demonstrated notable neurological improvement. She was more alert, oriented, and able to engage in full conversation. Her husband noted a marked reduction in confusion and apathy. Additionally, the patient was able to ambulate short distances with assistance and less hesitancy. Over the next 48 hours, she continued to be drained at scheduled intervals of 10-20 mL every two hours with no complications. Her clinical gains were sustained throughout the drainage period. She was evaluated by the physical therapy team and approved for inpatient rehabilitation (IPR) under the physical medicine and rehabilitation. Following drain removal on day three, the patient was discharged to IPR in stable condition with a plan for outpatient neurosurgical follow-up to evaluate candidacy for ventriculoperitoneal shunting on discharge from IPR.

## Discussion

NPH is a rare neurological condition that can be caused by excess CSF due to impeded flow or an increase in production [7]. It has an estimated prevalence of 3.7% among individuals 65 years and older [8], but it's reported to be 0.003% in patients under the age of 65 [9]. NPH is classically characterized by Hakim's triad of three classical symptoms of mental impairment, gait disturbance, and urinary or fecal incontinence.

Although 78-84% of patients have been reported to have the triad [10], some patients may have variable clinical symptoms [11]. Often, gait impairment is one of the first symptoms that patients will experience and is the most common overall as well [12]. The American Academy of Neurology guidelines mention that ventricular shunting can often improve patients with mild to moderate cognitive impairment and predominant gait disturbance [13]. However, certain studies indicate problems with incontinence can typically signify a later progression of the condition [14]. Specifically, this can be hard to quantify in this population as problems with frequency and urgency are common in the elderly [15] due to a multitude of reasons.

The majority of NPH is idiopathic in nature and often seen among the elderly. However, secondary NPH can affect individuals of all ages as causes can span meningitis, a brain tumor, traumatic brain injury, or even subarachnoid hemorrhage [4]. In our patient, secondary causes were thoroughly considered and excluded. There was no history of prior head trauma, intracranial surgery, subarachnoid hemorrhage, CNS infection, or neoplasm. Imaging revealed no evidence of mass lesion, prior hemorrhage, infarction, or post-traumatic changes. Although our patient's CSF profile showed elevated protein, low glucose, and increased nucleated cells, cultures and meningitis/encephalitis panels were negative, suggesting a nonspecific or reactive profile rather than infectious etiology. Mild pleocytosis and elevated protein have been reported in iNPH and in the absence of positive microbiologic or inflammatory markers, do not preclude the diagnosis [13].

Our patient's MRI demonstrated features consistent with idiopathic NPH (iNPH), including ventriculomegaly, periventricular hypoattenuation, and disproportionately enlarged subarachnoid spaces (DESH) [5]. These characteristic neuroimaging findings, combined with a compatible clinical picture and exclusion of known secondary causes, fulfilled established diagnostic criteria for probable iNPH. Our patient underwent continuous lumbar drainage, rather than a single tap test, and showed marked clinical improvement within 12-24 hours both cognitively and functionally. Such improvement after CSF diversion is regarded as one of the strongest prognostic indicators for shunt responsiveness [6]. Although shunt placement is the definitive treatment for NPH, in this case, the neurosurgery team deferred immediate ventriculoperitoneal (VP) shunting. Instead, the patient was discharged with outpatient follow-up to assess sustained improvement and determine long-term shunt candidacy. This is consistent with standard practice when the response to drainage is positive, but the clinical trajectory and long-term prognosis remain to be fully assessed.

Only a small number of early-onset iNPH cases (<60 years) have been reported in the literature. In a systematic review of over 3000 NPH cases, less than 2% were under age 60 [16]. Those younger patients tended to present with more headaches and fewer balance-related issues compared to older adults, which further complicates recognition. Our patient notably presented with classic triad features and no secondary cause, which reinforces the diagnosis of idiopathic NPH. This case highlights the need for clinicians to consider iNPH even in patients below the typical age threshold, particularly when both imaging and clinical presentation are strongly supportive. Future larger population-based studies should focus on uncovering further preferential clinical symptoms and potential genetic or environmental factors that may lead to early-onset presentation. Our case report is limited by the absence of a formal baseline cognitive assessment, which would have provided an objective measure of the degree of improvement following CSF diversion. However, multiple providers' notes consistently documented deficits in attention and short-term recall at presentation, with marked improvement in both domains after drainage. In addition, while CSF analysis showed mild abnormalities, infectious and inflammatory causes were excluded by negative cultures, meningitis/encephalitis panel, and neuroimaging. As with all single case reports, the findings may not be generalizable, but they underscore the importance of considering idiopathic NPH in younger patients presenting with classic features and supportive imaging.

## Conclusions

We describe a case of early-onset idiopathic NPH in a patient under 60 successfully managed through coordinated care between neurology, neurosurgery, and interventional radiology. This case underscores the value of prompt recognition, appropriate imaging, and multidisciplinary collaboration in optimizing outcomes. Clinicians should remain mindful that iNPH, though uncommon in this age group, should be considered when younger patients present with the characteristic triad and supportive imaging findings.
